# A Framework for Spatial Interaction Analysis Based on Large-Scale Mobile Phone Data

**DOI:** 10.1155/2014/363502

**Published:** 2014-11-04

**Authors:** Weifeng Li, Xiaoyun Cheng, Zhengyu Duan, Dongyuan Yang, Gaohua Guo

**Affiliations:** Key Laboratory of Road and Traffic Engineering of the Ministry of Education, 4800 Cao'an Road, Tongji University, Jiading District, Shanghai 201804, China

## Abstract

The overall understanding of spatial interaction and the exact knowledge of its dynamic evolution are required in the urban planning and transportation planning. This study aimed to analyze the spatial interaction based on the large-scale mobile phone data. The newly arisen mass dataset required a new methodology which was compatible with its peculiar characteristics. A three-stage framework was proposed in this paper, including data preprocessing, critical activity identification, and spatial interaction measurement. The proposed framework introduced the frequent pattern mining and measured the spatial interaction by the obtained association. A case study of three communities in Shanghai was carried out as verification of proposed method and demonstration of its practical application. The spatial interaction patterns and the representative features proved the rationality of the proposed framework.

## 1. Introduction

**Figure 1:**
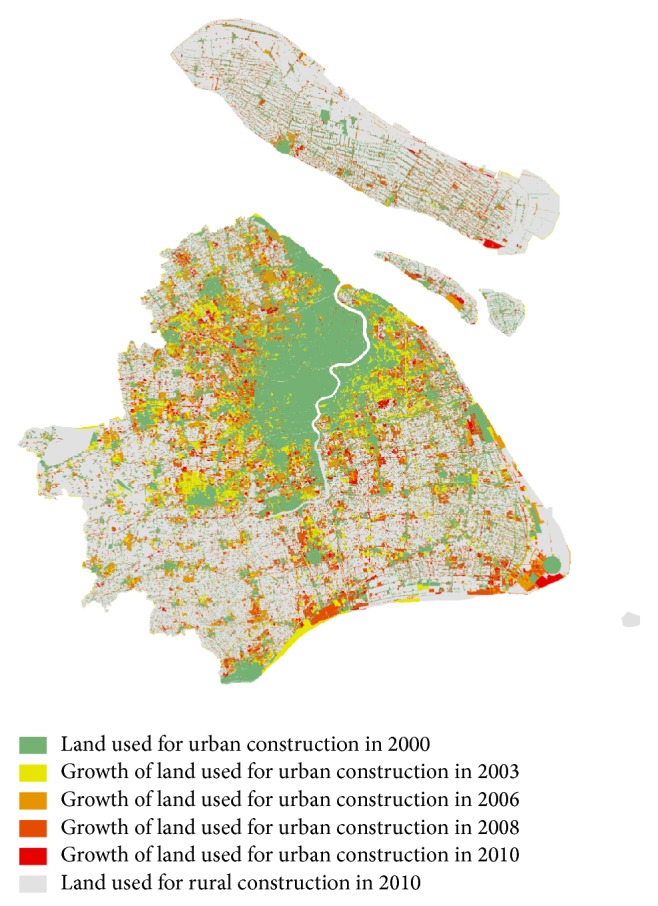
The process of urban sprawl in Shanghai.

Shanghai, the representation of mega cities in China, has been undergoing unprecedented urban sprawl. According to the official statistics, the land used for urban construction had almost doubled in the first decade of the 21st century, as shown in Figure 1. The rapid urban expansion also had significant effect on the trend of travel patterns. Particularly, the daily person trips and the average trip length were estimated to go through a rapid growth in the next decade. In the unparalleled process of urban sprawl, planners and operators seek access to the exact knowledge of interaction between individual behavior, urban space structure, and public transport service. However, the past experiences and traditional theories seem inadequate for the thorny situation.

Thanks to the new technology of data collection and the novel concept of big data, positive prospects for the solution to these issues can be seen. The newly arisen data sources enable the overall understanding in a large scale. In this paper, mobile phone data was used to analyze the spatial interaction. A novel framework that was compatible with the peculiar characteristics of mobile phone data was proposed.

Mobile phone data refers to the mobile connectivity logs collected by mobile operators [1]. It is a newly arisen dataset that can pervasively track people's movement in the spatiotemporal dimension [2]. Mobile phone data has been applied in many travel surveys as the supplementary data source for its huge volume, wide coverage, real-time production, automated collection, and low cost. Existing studies have also provided a series of approaches to the application of mobile phone data in traffic analysis [3, 4] and individual behavior analysis [5–8]. However, because of the peculiar characteristics of mobile phone data and the limitations of analysis technologies, the complete description of individual trajectories and the extraction of single trips from the continuous trajectories are not easily accessible based on mobile phone data alone. Thus, the compatibility as well as transplantability of traditional methodologies in the novel dataset is worth discussing.

Spatial interaction is defined as the process whereby entities at different location make contacts, demand/supply decisions, or locational choices [9]. Spatial interaction represents the potentiality of people to reach the opportunities in urban areas [10]. The comprehensive knowledge of spatial interaction in the different location of the city is the premise and foundation of urban planning and transportation planning in the new phase of urban construction. The conventional approach for spatial interaction analysis is carried out based on the spatial interaction models, such as gravity models, potential models, and retail models [11–13]. The basic assumption of these models is that the interaction is a function of the attributes of trip origins, the attributes of trip destinations, and the friction of trip distance.

Two points are to be emphasized here. Firstly, the input of the traditional models includes land use and impedance matrix; and they are usually gone and static. It is not able to describe the overall situation and reflect the dynamic evolution process, particularly in the rapid development of Chinese metropolises. Secondly, the traditional framework based on the concept of trip underlies the traditional spatial interaction analysis. The disadvantage in single trip extraction makes it inappropriate to transplant the traditional framework into the mobile-phone-based analysis. With the introduction of association analysis, a basic framework for spatial interaction analysis based on the incomplete trajectory information was proposed in this paper. The framework adopted frequent pattern mining and measured the spatial interaction by the obtained association.

The rest of the paper was organized as follows: (a) the overview of GSM network and the database schema of mobile phone data were introduced in the next section; (b) a three-stage framework for spatial interaction analysis based on mobile phone data was described in the third part; (c) the case study of three communities in Shanghai was carried out to verify the proposed framework and demonstrate the practical application; (d) conclusions and future directions were given in the last paragraph.

## 2. Preliminaries

### 2.1. Overview of the GSM Network

GSM network is a world-wide wireless network of mobile communication with an extensive coverage. To establish the point-to-point connections, the organizational structure of GSM network is divided into several processing elements [14].

Cells are the smallest units of a GSM network, each stretched out by the radio coverage of a base transceiver station (BTS). Mobile stations (MS, the terminal devices) get access to the whole backbone network of the GSM network through the radio link to the BTSs.

Normally, the mobility management layer only identifies a limited number of cells in which the MS is located. This group of cells form a location area (LA) and comprise the lowest existing level of the location information. Each LA is identified by a unique location area identifier (LAI) and each cell in the LA by a cell identifier (CI). The combination of LAI and CI can uniquely identify the BTS in a GSM network.

The GSM system tracks the status of MSs and allows calls, SMS, and other services to be delivered to them. If some specific communication procedures are detected, the system will be informed to register the updates in the database. The specific procedures include IMSI (International Mobile Subscriber Identification) attach, IMSI detach, roaming, location update, periodical location update, and so on.

### 2.2. Overview of the Mobile Phone Dataset

**Figure 2:**
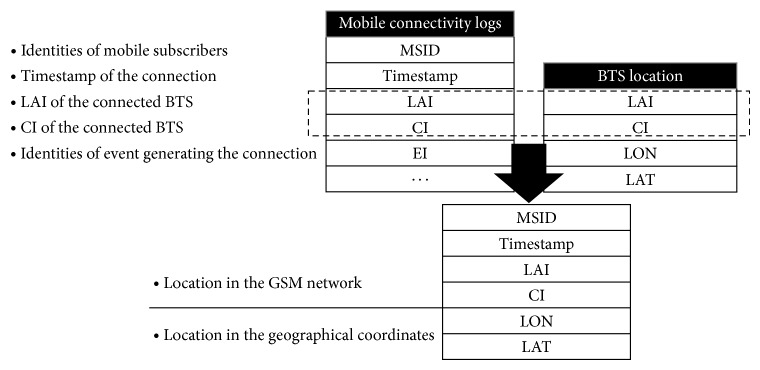
Schema of the original dataset.

Mobile phone data used in this paper was collected for billing and operational purposes during September 2011 throughout Shanghai. The market share of the carrier involved was more than 70% in 2011, which was large enough to ensure the statistical significance of the following analysis in this paper. Two data tables composed the original dataset, including the basic connectivity information of MSs and the location information of BTSs. In the original dataset, the daily connectivity logs are no less than 100 GB. 0.7 billion connectivity logs from more than 17.5 million MSs are collected on an average day. The dataset schemata presenting the relationship between the two data tables were illustrated in Figure 2.

The mobile connectivity table stores the logs of connection between MSs and BTSs. Fields of the table include the identities of mobile subscribers, the LAI and CI of the connected BTS, the identities of event generating the connection, and other fields representing the communication patterns. The BTS location table comes from the mobile carrier in a top-down manner and stores the geographical coordinates of BTSs in longitude and latitude.

Through the relational operation, with LAI and CI acting as match fields, mobile subscribers' activities in the GSM network were mapped onto the geographical coordinates.

## 3. Methodology

The aim of this study was to explore an approach for spatial interaction analysis based on the mobile phone data. However, the raw data collected in the mobile cellular communication is not applicable to the transportation-related analysis. The main obstacles lie in the incompatibility of original data structure in the traffic analysis, the correspondence between virtual activities and physical activities, and the appropriate measurement of spatial interaction. For reasons mentioned above, a three-stage model was proposed to overcome the obstacles and construct the framework for spatial interaction analysis.

- *Stage 1: Reorganization of Original Dataset*. Data preprocessing to transform the original communication logs to a simpler data structure suitable for modeling.
- *Stage 2: Identification of Activity Points*. Extraction of the critical anchor points in people's daily trajectories.
- *Stage 3: Measurement of Spatial Interaction*. Data mining for the interaction between different areas based on the spatial distribution of activity points and the association obtained through frequent pattern mining.

In the following sections, the three stages in the framework were discussed in detail and their specific procedures were described by pseudocode, respectively. Finally, the overall structure of the three-stage framework was given in the last section.

### 3.1. Reorganization of Original Mobile Phone Data

Since the mobile phone data was collected for communication industry, it was not primarily designed for modeling purposes and not in an easy-to-use format. Particularly, the peculiarity of mobile phone data collection makes it unfit for the spatial and statistical analysis as well as the visualization of data mining results. To make up the deficiencies, binning method and raster data structure were introduced in this study.

#### 3.1.1. Binning Method

Overlaps exist in the coverage areas of two adjacent BTSs. In particular, coverage radius of BTS in the central city of Shanghai is only 500~800 meters on average. Frequent handover may occur as the MS enters the overlaps of the serving cell and the adjacent cells. The frequently gratuitous handovers lead to the data noise and the waste of system resources. Binning method was used in this study to smooth the location information and reduce the volume of data.

The chronologically sorted logs were distributed into bins of equal width in the temporal dimension. All the logs in the same bin were replaced by one equivalent log. The timestamp of the equivalent log was the bin median; and the location information was replaced by the weighted average of the original coordinates in the same bin. Let the width of each bin be 10 minutes; the specific procedure was described in Algorithm 1.

Since the frequent handover was represented in the original data as a cluster of logs in an incredibly short period of time, the negative effect of frequent handover was eliminated by assigning small weights to logs with small intervals. What is more, with one equivalent log acting as alternative for all the actual logs in a certain bin, the volume of data was reduced sharply.

The selections of bin width value as well as the accuracy of mining results obtained with the binned data are to be discussed in the forthcoming articles.

#### 3.1.2. Raster Data Structure

By 2011, 23,918 BTSs distributed unevenly and irregularly throughout Shanghai. The data structure was unfit for the spatial and statistical analysis, the mining results visualization, and the further data fusion with other data sources. The raster data structure was applied for the transformation of BTS's geographical coordinates.

**Figure 3:**
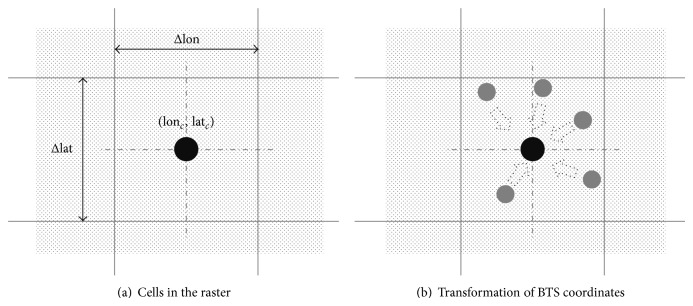
Illustration of raster data structure.

In this study, a raster was constructed to cover the city territory of Shanghai. For the facility of calculation, cells of the raster were delimited with meridians and parallels in fixed intervals. Therefore, each cell could be determined by the coordinate of centroid (lon_*c*_, lat_*c*_) and the length of sides Δlon, Δlat, as shown in Figure 3. With reference to the average radio coverage of BTS and the required spatial scale in traffic analysis, the size of cells was set as 500 meters by 500 meters. All the BTSs in the same cell were replaced by one equivalent BTS coordinated at the cell's centroid.

The calculation of the four critical parameters and the transformation of BTSs' geographical coordinates were described in Algorithm 2.

The city territory of Shanghai was covered by the raster with 245 rows and 348 columns. In the output of the algorithm, the 23,918 actual BTSs throughout Shanghai were reduced to 10,303 equivalent BTSs.

### 3.2. Identification of Activity Points

The original mobile phone data describes the individual's virtual activities and provides the basic information of time, location, and frequency. The synthesis and summarization of this basic information enable the inference of physical activities and the accessibility to the individual behavior patterns.

In this study, the activity point was defined as the location at which a certain mobile subscriber continuously stayed for no less than 30 minutes. Activity points act as critical anchor points in people's daily trajectories, incorporating home and workplace as two particular kinds of activity points. A set of activity points arranged in chronological order formed the activity chain of a certain mobile subscriber. The identification of activity points can be carried out as Algorithm 3.

### 3.3. Measurement of Spatial Interaction

The macroscopic zonal interaction can be obtained through the aggregate analysis of activity chains. In the existing models, the spatial interaction is analyzed based on the concept of trips. However, as for mobile phone data, the extraction of single trips from the continuous daily trajectories is not easily accessible. Though the particular data processing may contribute to the relatively accurate trip identification, the extra operation is doomed to lower the efficiency of mass data mining. In this study, the novel approach for spatial interaction analysis was proposed based on frequent pattern mining. The correlations and associations between different areas were applied to measure the spatial interaction.

Frequent pattern is item sets that appear in a dataset with frequency no less than a user-specified threshold. In this study, identities of areas acted as item sets; and each transaction was a sequence of area identities obtained from the activity chain of a certain mobile subscriber. Concretely speaking, let *M* = {*m*_1_, *m*_2_,…, *m*_*N*_} be an item set, where *m*_*i*_, *i* = 1,2,…, *N*, represent the identity of the *i*th area. With the specific mapping relation between areas and geographical coordinates, the activity chain *A* could be converted to a sequence of area identities AI. Therefore, given the set of study objects *U*, AI^*u*_*i*_^, *u*_*i*_ ∈ *U*, was one of the transactions and comprised the input dataset of spatial interaction calculation.

Let *X*, *Y* be sets of items, where *X* ⊂ *M*, *Y* ⊂ *M*, *X* ≠ *⌀*, *Y* ≠ *⌀*, and *X*∩*Y* = *⌀*. *P*(*X* ∪ *Y*), the probability that a converted activity chain contained the union of sets *X* and *Y*, represent the association between areas in *X* and *Y* as well as the spatial interaction of the areas in the two sets.

The acquisition of input database and the measurement of spatial interaction were performed as Algorithm 4. It should be noted that the following pseudocodes lay emphasis on the introduction to the conversion from activity chains into sequences of activity identities. The frequent pattern mining was carried out through the commonly used algorithm of Frequent Pattern Growth (FP-growth), which was unnecessary to go into details.

### 3.4. Overall Structure of the Three Stages

**Figure 4:**
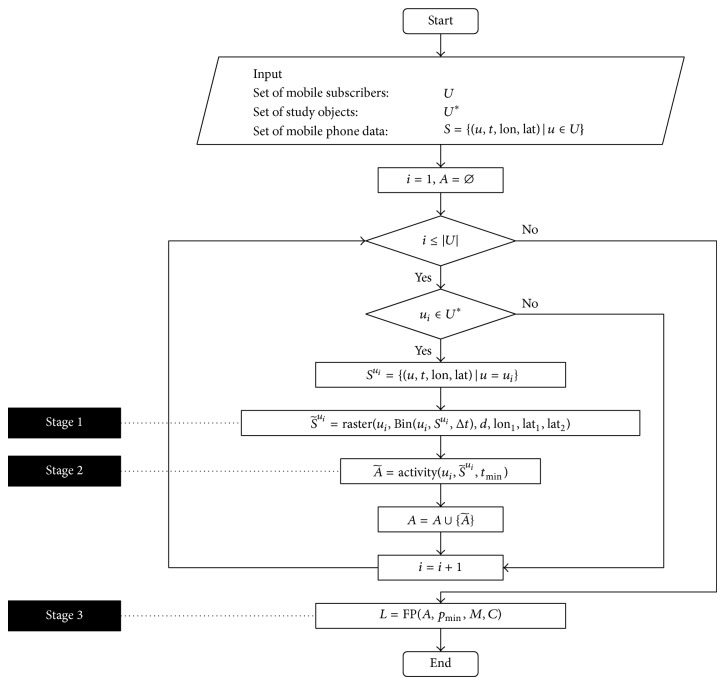
Framework for spatial interaction analysis.

With the introduction of the three stages mentioned above, the framework for spatial interaction analysis based on mobile phone data can be organized as in Figure 4.

## 4. Case Study

### 4.1. Study Areas

**Figure 5:**
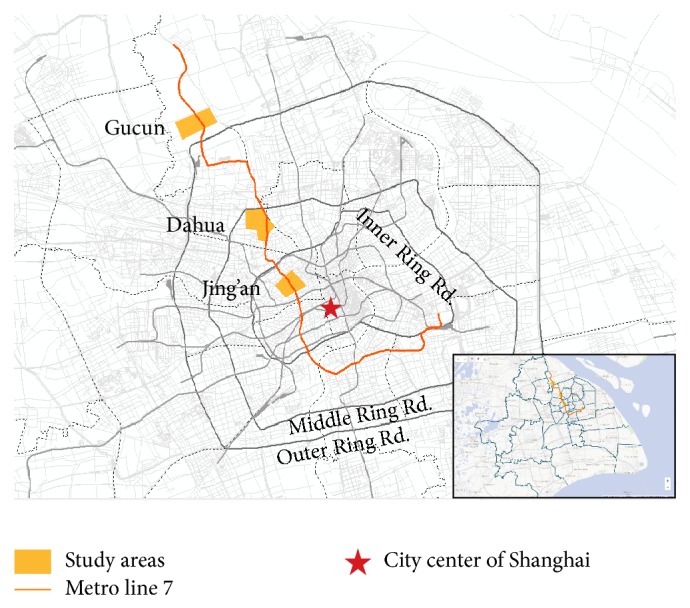
Study areas.

To demonstrate the practical application of the analysis framework proposed in this paper, three communities were selected as study areas, as shown in Figure 5. The three study areas were selected from the communities along Metro line 7 in Shanghai with the overall consideration of data quality, construction history, built environment, location, and resident population. The three selected communities are Jing'an, Dahua, and Gucun. Generally speaking, Jing'an, Gucun, and Dahua are, respectively, the typical representatives of the mature communities in the city center, the newly constructed communities in the suburbs, and the communities in between. The three study areas are illustrated in Figure 4; and the key information of the three study areas is listed and compared in Table 1.

### 4.2. Study Objects

Residents in the study areas were considered as the study objects. Method of mobile-phone-based resident identification proposed in our previous research [16] was introduced to determine the study objects. A certain mobile subscriber could be labeled as the resident in the study area if the criteria were satisfied that the mobile subscriber once stayed in a certain study area for no less than 6 hours during the time period from 9 p.m. to 6 a.m, frequency of which exceeded 20 in a month.

As a result, there were 1,363 residents identified in Gucun, 2,955 in Dahua, and 14,901 in Jing'an. *U*_1_^*^, *U*_2_^*^, and *U*_3_^*^ denoted the sets of residents in the three study areas, respectively, and acted as the input parameters in the spatial interaction analysis.

### 4.3. Results and Discussion

#### 4.3.1. Activity Points

**Figure 6:**
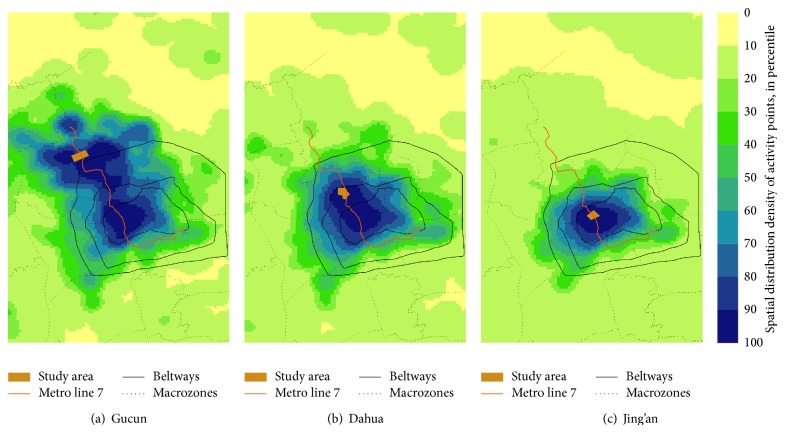
Spatial distribution of activity points.

Activity points are the intermediate results of the spatial interaction analysis.  Figure 6 illustrates the spatial distribution patterns of the activity points extracted from the residents' daily trajectories. The spatial distribution of activity points depicts the fundamental state of spatial interaction.

#### 4.3.2. Spatial Interaction

With reference to the Shanghai Fourth Comprehensive Traffic Investigation, the city territory of Shanghai was divided into 35 traffic macrozones. The identities of the 35 macrozones and the identity of the study area together constituted the item set *M* in the frequent item set mining. The minimum support threshold *p*_min⁡_ was set to be 2%.

**Figure 7:**
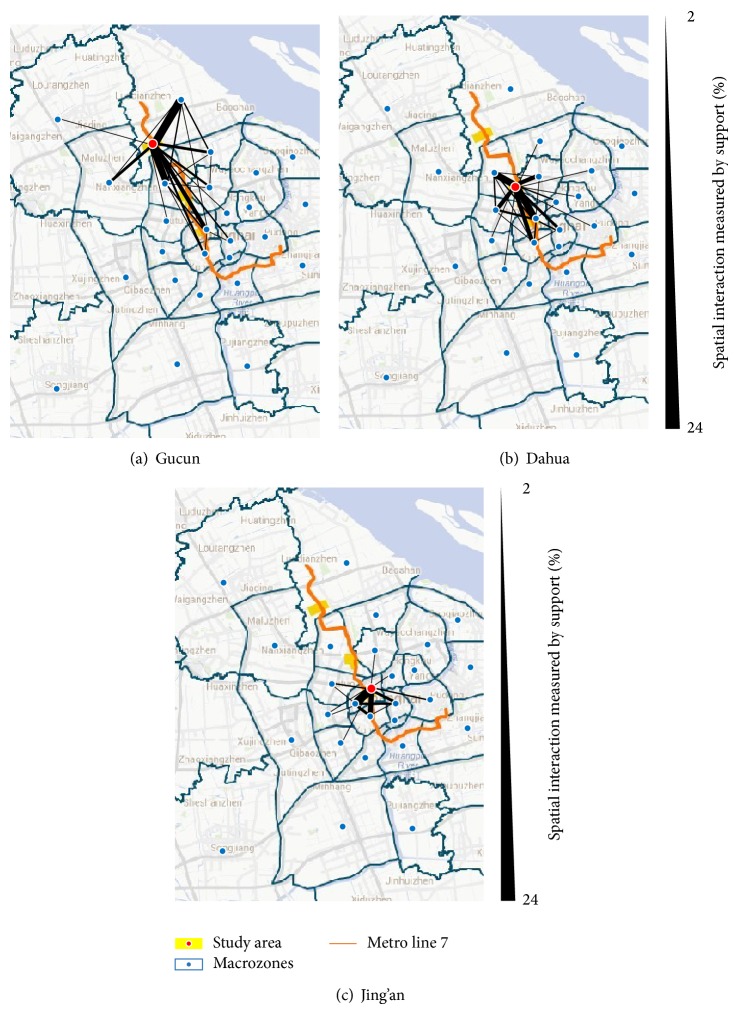
Spatial interaction of residents' activities in the study areas.

The spatial interaction of residents' activities is fetched from the outputs. The frequent 1-item sets depict the spatial distribution of activity points in different macrozones, which yields a similar result as Figure 6.  Figure 7 illustrates the outcomes of frequent 2-item sets and shows the spatial interaction between two different macrozones.

#### 4.3.3. Discussion

Through the visualization of calculation outcomes, a brief analysis can be carried out to discover some representative features in spatial interaction.

As shown in Figure 6(a), the spatial distribution of Gucun residents' activities is a nonuniform distribution shaped like a binuclear dumbbell. There are two centers of activity: the regional center nearby and the area in the central city along Metro line 7. As shown in Figure 7(a), both of the two centers have strong association with the surrounding areas. There also exists a strong link between the two activity centers, which plays the role of handle that joins the centers.

Figure 6(b) shows a less centripetal tendency for the residents' activities in Dahua. The spatial distribution of residents' activities shapes like a ribbon along Metro line 7. However, as Figure 7(b) illustrates, there are still two activity centers. Due to the short distance between Dahua and the central city, the two activity centers are closely interlinked and fuse to form one morphologically. But from the viewpoint of function level, they are still divergent.

The activities of residents in Jing'an distribute evenly without evident centralization, characterized by the flexible shape and the uniform distribution in Figure 6(c). The spatial interaction in Figure 7(c) only shows the strong associations between Jing'an and the surrounding areas.

The above analysis proves the rationality of the framework proposed in this paper. The long-term and pervasive monitoring of activities based on mobile phone data is an effective way to obtain the spatial interaction between the different areas. The representative features extracted can be applied in the further studies on the interaction between individual behavior and urban space structure.

## 5. Conclusion

Mobile phone data can pervasively track individual behavior in both temporal and spatial dimension. However, for many reasons, the conventional framework for spatial interaction analysis is not applicable to the mobile-phone-based analysis. A framework was proposed for the compatibility with the peculiar characteristics of mobile phone data. There were three stages in the proposed framework. Stage 1 provided an approach to preprocess the original dataset through binning method and raster data structure. Stage 2 aimed at the activity point extraction from the individual's daily trajectories. The last stage was to measure the macroscopic zonal interchange through the frequent item set mining. In the case study of the three communities in Shanghai, spatial interactions of residents' daily activities were obtained through the proposed framework. In the brief analysis of the outputs, the mobile-phone-based analysis was proved an effective way to analyze the spatial interaction and extract the representative features.

Nowadays, open data has become one of the central topics of city development. The novel datasets are considered as one of the effective ways to understand the rapid development in Chinese mega cities. The data mining of public data and the data fusion with other data sources will become the key technologies in urban planning and transportation planning. The mobile phone data is one of the newly arisen datasets. However, it is still blank in the systematic theory and detailed study at the application of mobile phone data in traffic analysis. The variety of data processing makes it extremely difficult to the further studies in data fusion and data mining. The three-stage framework proposed in this study is the first step to set up the platform for the standardized and normalized framework of mobile phone data analysis.

## Figures and Tables

**Algorithm 1:**
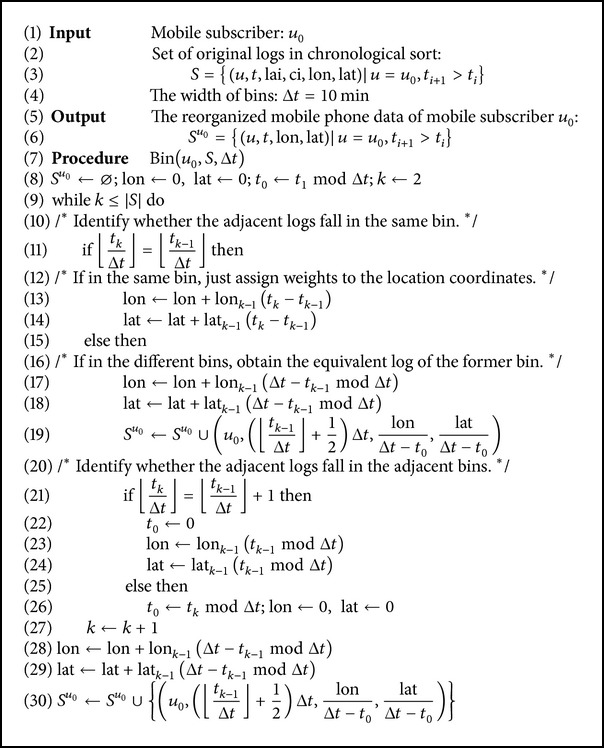
Binning method of original mobile phone data.

**Algorithm 2:**
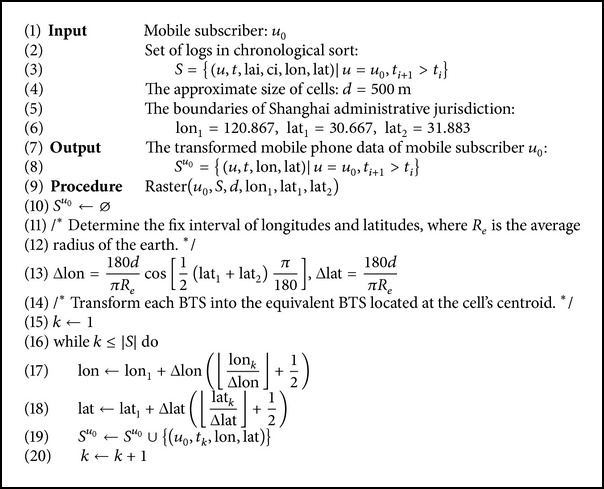
Transformation of geographic coordinates.

**Algorithm 3:**
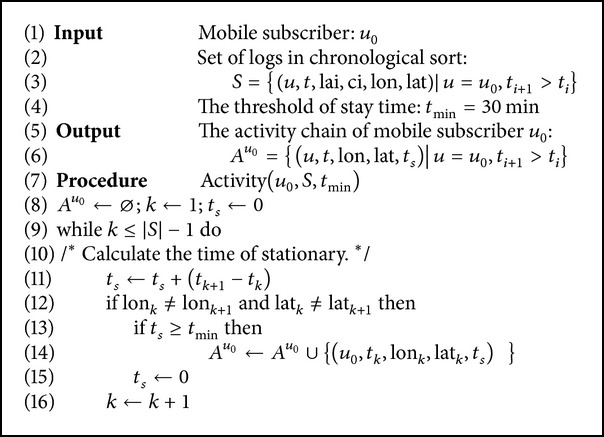
Identification of activity points.

**Algorithm 4:**
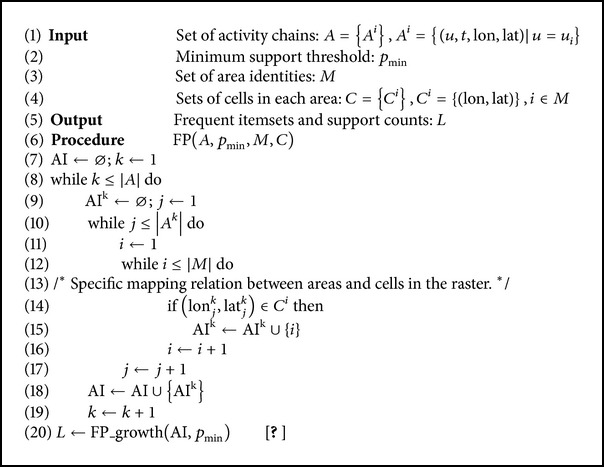
Measurement of spatial interaction.

**Table 1:** Key information of study areas.

	Area (km^2^)	Distance to city center (km)	Location	Households^*^
Gucun	4.50	16.71	Outside the Outer Ring Road	67,860
Dahua	3.44	7.46	Between the Inner Ring Road and the Middle Ring Road	43,095
Jing'an	2.56	3.16	Inside the Inner Ring Road	39,423

^*^Statistics by 2011.
